# Production of Silver Nanoparticles with Strong and Stable Antimicrobial Activity against Highly Pathogenic and Multidrug Resistant Bacteria

**DOI:** 10.1155/2014/704708

**Published:** 2014-06-30

**Authors:** Amr T. M. Saeb, Ahmad S. Alshammari, Hessa Al-Brahim, Khalid A. Al-Rubeaan

**Affiliations:** ^1^Strategic Center for Diabetes Research, King Saud University, P.O. Box 18397, Riyadh 11415, Saudi Arabia; ^2^The National Nanotechnology Center, King Abdulaziz City for Science and Technology, P.O. Box 6086, Riyadh 11442, Saudi Arabia; ^3^University Diabetes Center, King Saud University, P.O. Box 18397, Riyadh 11415, Saudi Arabia

## Abstract

*Aims*. To synthesize, characterize, and analyze antimicrobial activity of AgNPs of *Escherichia hermannii* (SHE), *Citrobacter sedlakii* (S11P), and *Pseudomonas putida* (S5). *Methods*. The synthesized AgNPs were examined using ultraviolet-visible spectroscopy (UV-vis) and, zeta potential, and the size and the morphology obtained from the three different isolates were also confirmed by TEM. *Results*. Among the three isolates tested, SHE showed the best antimicrobial activity due to the presence of small (4–12 nm) and stable (−22 mV) AgNPs. Stability of AgNPs was also investigated and found to be dependent on the nature of isolates. *Conclusion*. Produced AgNPs showed particle stability and antimicrobial efficacy up to 90 days of production. Our AgNPs exhibited greater antimicrobial activity compared with gentamicin against *P. aeruginosa* isolates and vancomycin against *S. aureus* and MRSA isolates at very low concentration (0.0002 mg per Microliters).

## 1. Introduction

Multidrug resistance is a growing problem all over the world. The widespread and uncontrolled use of antibiotics has led to resistance to conventional antimicrobial agents for many bacterial pathogens and poses a major health threat. For instance, more than 25% of insidious staphylococcal isolates are methicillin resistant* Staphylococcus aureus* MRSA in some countries [1]. Silver nanoparticles have been known to exert inhibitory and bactericidal effects and to possess a broad spectrum of antimicrobial activities against many Gram-positive, Gram-negative [2–4], and fungal pathogens [5].

Metal nanoparticles (NPs) (e.g., silver, gold, platinum, etc.) have been the subject of ample research interest in the last few years, due to their exciting size-dependent electrical, optical, physical, and chemical properties. In particular, colloidal silver nanoparticles (AgNPs) have attracted increasing research attention in the field of microbiology [6–8]. Since the properties of the AgNPs depend significantly on their size and morphology, intensive investigations have focused on the control of such parameters, using different preparation methods [9]. Numerous methods have been described for the preparation of colloidal AgNPs, such as conventional chemical reduction [10], radiation chemical reduction [11], sonochemical [12], and electrochemical reduction [13]. The size of AgNPs prepared using these methods are found to be in the range of 5 nm to 40 nm [14, 15]. In addition, a stable AgNPs can be produced by electrostatic complexing of silver ions using an anionic surfactant aerosol [16]. The major problem of these methods classified as chemical and physical methods is that they are expensive and dangerous and could present environmental and biological hazards. Furthermore, metal particles produced by these methods usually carry traces of toxic chemicals, which restrict their use in clinical and human applications [16]. Therefore, there is a need to synthesize AgNPs using ecofriendly, clean, and more effective methods against human pathogens.

In response, biological method of preparing metal nanoparticles (e.g., AgNPs) seems to be an interesting route for green chemistry. In this regard, several microorganisms including bacteria, yeasts, and fungi have been used [17–19]. Many bacterial species have been used for the production of different sizes of silver particle, including* Lactobacillus* strains 500 nm [20],* Bacillus megaterium* 46.9 nm [19],* Klebsiella pneumonia* 50 nm [20],* Bacillus licheniformis* 50 nm [21],* Corynebacterium* sp. 10–15 nm [22],* Escherichia coli* 1–100 nm [23, 24],* Proteus mirabilis* 10–20 nm [25],* Bacillus *sp. 5–15 nm [26],* S. aureus* 1–100 nm [27], and* Pseudomonas aeruginosa* 13 nm [28].

To the best of our knowledge, this is the first report to show the production of AgNPs using the supernatant of three new species collected from the Saudi environment. The synthesized nanoparticles were characterized by different methods such as UV-vis, zeta potential, and TEM. The colloidal AgNPs were also evaluated for antimicrobial activity against Gram-positive and Gram-negative pathogens.

## 2. Material and Methods

### 2.1. Microbial Strains and Culture Conditions

Bacterial isolates were extracted from soil samples, which were collected from different geographical areas in Riyadh, Kingdom of Saudi Arabia. Such areas have been known to be contaminated with traces of metal, that is, the old industrial area, the new industrial area, Khazzan area, and Al-Shimasi area. The bacteria were isolated by plating dilutions of soil samples in saline solution (0.9% NaCl) on nutrient agar and incubated at 28°C for 48 h. Individual colonies of bacteria which varied in shape and color were chosen and purified by streaking on nutrient agar. The bacterial isolates were identified using the VITEK 2 GN ID and VITEK 2 system. A total of 35 bacterial isolates were collected, purified, and identified to the species level. Of the 35 bacterial isolates, three isolates were selected for the current study, namely,* Escherichia hermannii* (isolate SHE),* Citrobacter sedlakii* (isolate S11P), and* Pseudomonas putida* (isolate S5).* Pseudomonas* selective medium (cetrimide agar) was used in isolation and identification of* Pseudomonas* isolates. Ethylene Methylene Blue (EMB) medium was used to isolate and verify bacterial isolates belonging to Enterobacteriaceae.

### 2.2. Preparation of Bacterial Cell Free Extract

*E. hermannii*,* C. sedlakii*, and* P. putida* isolates were cultured in nutrient broth medium to produce the bacterial biomass. The culture tubes (Corning Incorporated, 430290, New York, USA) were incubated on orbital shaker (Thermo Scientific, MAXQ 4000, USA) at 28°C overnight at 220 rpm. The biomass was harvested by centrifugation 10000 rpm for 10 minutes. The supernatants were collected for the production of silver nanoparticles.

### 2.3. Biosynthesis and Tentative Characterization of Silver Nanoparticle

Silver nitrate (AgNO_3_) (D.F. Goldsmith Chemical and Metal Corp.) at the concentration of 10^−3^ M (1%V/V) was added to the reaction vessels containing the bacterial isolate supernatants. The reduction of silver ions was carried out in the bright conditions for 24 hours. In addition, the blank test was also performed in the absence of silver nitrate. The bioreduction of (Ag^+^) ions was observed by monitoring changes of the color from light brown to dark brown and was observed among tested isolates and the absorption was periodically measured using UV-spectrophotometer (2800 UV-vis, UNICO, NJ, USA) using quartz cuvette.

### 2.4. Characterization of Silver Nanoparticles

#### 2.4.1. Ultraviolet-Visible Absorption Spectroscopy (UV-Vis)

The optical properties (absorbance) of colloidal sliver nanoparticles solutions were acquired with a (*UV-vis*) spectrometer (Avantes-2048, light source combined deuterium-halogen). The spectra were collected over a range of 200–1100 nm (with an optical path length of 0.4 cm) as a function of reaction time by directly dipping the optical probe in the reaction vessel.

#### 2.4.2. Transmission Electron Microscopy (TEM)

Size analysis of colloidal AgNPs was characterized using TEM JEM-2100F at a voltage of 200 kV. The samples were prepared by mounting a drop of the aqueous suspension containing the AgNPs on a carbon grid, which then was placed on filter paper to absorb excess solvent. The average particle diameter and size distribution were calculated using Java image tool software (ImageJ), based on the data of an average of 70–100 particles.

#### 2.4.3. Antimicrobial Susceptibility Test

Antimicrobial activities of biosynthesized silver nanoparticles from SHE, S11P, and S5 isolates were tested against the following strains:* Pseudomonas aeruginosa* ATCC 27853,* Escherichia coli* ATCC 25922,* Klebsiella pneumonia* ATCC 700603,* Staphylococcus aureus* ATCC 29213, and* Staphylococcus epidermidis* ATCC 12228 using the agar diffusion assay method [29] using 20 *μ*L of sample nanoparticles solution (0.0002 mg). Briefly, bacterial lawns of bacterial test strains and hospital isolates were made on readymade Müller-Hinton agar and left to dry. The sterile discs were loaded with 20 *μ*L silver nanoparticles solution containing approximately 0.0002 mg silver nanoparticles and then placed on Müller-Hinton agar nutrient agar plate with bacterial lawns and incubation at 37°C for 24 hours. All experiments were done in aseptic condition in laminar air flow cabinet. Zones of inhibition for control, SNPs, and antibiotics were measured in millimeters. The drug of choice for each tested strain was used for comparison, namely, gentamicin (*P. aeruginosa*), ceftriaxone (*E. coli* and* K. pneumonia*), and clindamycin (*S. aureus* and* S. epidermidis*). The zones of inhibition were measured, and the mean value was reported in millimeters. In order to test the efficacy of biosynthesized silver nanoparticles against real life pathogens, we tested the SHE silver nanoparticles against the major pathogens of Diabetic Foot Ulcer (DFU) isolated from diabetic foot patients recruited from University Diabetes Center in Riyadh, the capital city of Saudi Arabia, by using the agar diffusion assay method [29]. Many studies have found that the most difficult to treat pathogens of DFU are either Gram-negative pathogens, namely,* P. aeruginosa,* or Gram-positive pathogens, namely,* S. aureus*, especially methicillin-resistant* Staphylococcus aureus* (MRSA). We investigated antimicrobial activities of biosynthesized silver nanoparticles against 22 isolates of* P. aeruginosa*, 21 isolates of* S. aureus* and 22 isolates of MRSA. We used gentamicin against* P. aeruginosa* isolates and vancomycin against* S. aureus* and MRSA isolates and were compared with our prepared silver nanoparticles. In addition, we studied the synergistic effect of combining silver nanoparticles and the antibiotic against the pathogenic organisms. The zones of inhibition were measured, and the mean values were reported in millimeters. The mean zone of inhibition ± SD was calculated and *t*-test was used to calculate *P* values among different treatment groups using SPSS software version 17.

## 3. Results and Discussion

The physicochemical properties of AgNPs obtained by different isolates, namely,* Escherichia hermannii* (SHE),* Citrobacter sedlakii* (S11P), and* Pseudomonas putida* (S5) were carried out using several methods such as (*UV-vis*) absorbance spectroscopy, Transmission Electron Microscopy (TEM), and zeta potential, as described below.

### 3.1. Optical Observation

**Figure 1:**
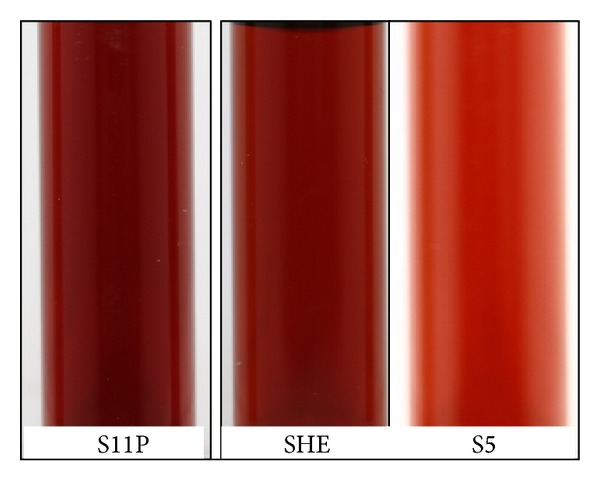
Supernatants of* C. sedlakii* (S11P),* E. hermannii* (SHE), and* P. Putida* (S5) after the addition of 1 Mm of silver nitrate and incubation for 24 hr in light at room temperature.

The reduction of Ag ions to Ag metal nanoparticles may be optically approved by color changes of the starting materials from colorless to brown. Color change observed is an indication of the formation of colloidal AgNPs [30]. However, we point out that slight color changes occurred, which might be due to the variation in the nature of isolates and size and shape of the metal particles. Moreover, that slight color changes occurred could also be due to the difference in the relative activity in reduction of silver nitrate ions to metal nanoparticles due to the nature produced proteins. Though, it can also reflect the nanoparticles surface plasmon resonances (SPRs). The simultaneous mixing of silver nitrate and different isolate solutions led to the formation of colloidal Ag metal nanoparticles by the appearance of the typical brownish color of the final slurry, as shown in Figure 1. It is clear from Figure 1 that colloidal AgNPs prepared using SHE and S11P showed brownish color which indicates that such isolates produced AgNPs with similar particle size. In contrast, AgNPs prepared using S5, as isolate, exhibited a light brown color, which gives a clue that the particle size is dissimilar to those prepared by SHE and S11P. Such observation indicates that the three isolates used here reduced the silver ions to silver nanoparticles with different sizes, which can be confirmed by (*UV-vis*) and TEM. Such colors result from a SP resonance initiated by the interaction of the electric field of visible light with the confined electron gas within the particles, which caused collective oscillation of the conduction electrons with respect to the core [31, 32].

### 3.2. UV-Vis Spectroscopy

**Figure 2:**
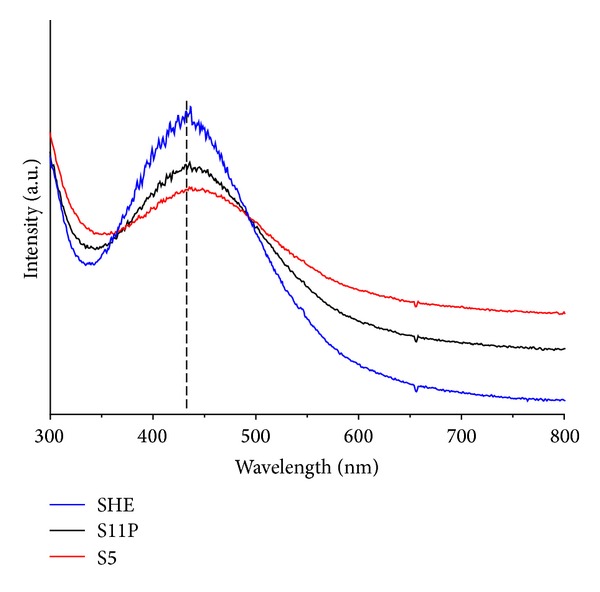
UV-vis spectroscopy absorption spectra of silver nanoparticles synthesized by different isolates (SHE, S11P, and S5 cultures).

UV-vis spectroscopy is one of the most useful methods for characterizing the optical response of metal nanoparticles (e.g., AgNPs). Such a method has been proved to be quite sensitive to the formation of colloidal metal nanoparticles, due to their intense surface plasmon resonances (SPRs) [33]. Typically, the characteristic formation of colloidal silver nanoparticles is confirmed by the appearance of the sharp SPRs in the range of 350–600 nm [34, 35]. It should be noted that the position of SPRs depends on different factors (e.g., size, shape, etc.). UV-vis absorption spectra (Figure 2) of colloidal AgNPs obtained by the different isolates showed SP bands, which differ in their *λ*_max⁡_ and SP band intensities, indicating a clear influence of the nature of isolates on these parameters. The spectra were recorded when both the color and absorption intensity of the colloidal samples stayed constant. It is clear from Figure 2 that each isolate used shows a single and different SPRs position in the range of 400–500 nm. In addition, no SPRs were observed at more than 500 nm, indicating that most of the AgNPs obtained have small size and similar shape. Such observation also gives preliminary indications regarding the size and size distribution of colloidal AgNPs [36, 37]. Among the three different isolates used, application of S5 showed plasmon band positions with a higher wavelength (446 nm) compared to SHE and S11P. This band was also observed to be somewhat broader and less intense. On the other hand, in case of using SHE and S11P as isolates, UV-vis results displayed that the SPRs becomes sharper and shifts towards lower wavelength (SHE = 438 nm, S11P = 441 nm), which indicates that the particle size of AgNPs decreased.

### 3.3. TEM Study for Fresh Colloidal AgNPs

**Figure 3:**
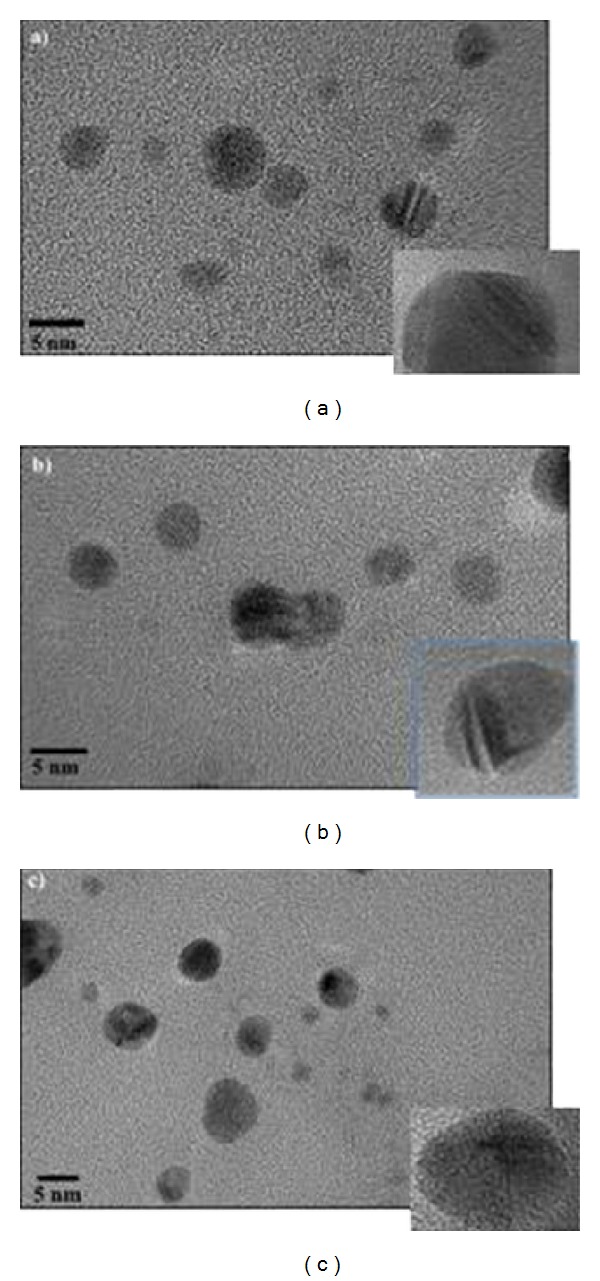
TEM image of silver nanoparticles biosynthesized by* E. hermannii* isolate SHE (a),* C. sedlakii* isolate S11P (b), and* Ps. putida* isolate S5 (c).

To understand the surface morphology and to provide more information about the size of colloidal AgNPs prepared by different isolates, TEM investigation was conducted under similar conditions, as shown in Figure 3. It can be seen from the shape of Ag nanoparticles obtained from SHE, S11P and S5 isolates are mostly spherical; and the average particle size is in the range of 4–30 nm. The TEM image of the sample using SHE and S11P (Figures 3(a) and 3(b)) gave corroborative evidence on the findings that SHE- and S11P-reduced silver colloids did not aggregate in solution, confirming the suitability of SHE and S11P culture supernatants not only as reducing agent, but also as an effective stabilizer. In contrast, the Ag nanoparticles were shown to be aggregated in case of using S5 as reductant. Among different isolates, SHE (4–12 nm) and S11P (4–15 nm) gave the smallest AgNPs with a narrow size distribution. In contrast, AgNPs prepared using S5 showed slightly larger particles, with broad size distribution (4–30 nm).

### 3.4. Zeta Potential Observations of Fresh Colloidal AgNPs

Zeta potential can be used to gain further insights into the stability of the obtained colloidal AgNPs. The magnitude of zeta potential gives an insinuation of potential stability of colloid. It should be noted that the particles with zeta potential values more positive than +30 mV or more negative than −30 mV are considered to be stable [38]. In contrast, the colloids are least stable at isoelectric point, where the zeta potential is zero. Herein, the *ζ* values varied in the range from −12 mV to −30.4 mV, depending upon the type of isolates applied. Thus, the different stability of colloidal AgNPs prepared by different isolates is also reflected by the changes in *ζ* values. Interestingly, the zeta potential value confirmed the high stability of the freshly prepared (mV_0_) colloidal mixture formed using S5 isolate (−30 mV), whereas the less stable particles were obtained with S11P and SHE isolates (−12.5 mV).

### 3.5. Long-Term Stability of the Colloidal AgNPs

The long-term stability of the colloidal AgNPs obtained by using different isolates, namely, SHE, S11P, and S5, was monitored over a longer time period by various techniques. The colloidal AgNPs were deliberately stored for this purpose in a closed glass vessel in dark environment. Afterwards, samples were taken at different predefined times (e.g., 1 day, 30 days, and 90 days) and were characterized by zeta potential measurements, UV-vis, and TEM to check the changes in zeta potential values, SP bands, size distribution, morphology, and so forth. The time-dependent changes using different isolates are summarized in Table 1 and described herein.

### 3.6. Optical Observations for Fresh Colloidal AgNPs

**Figure 4:**
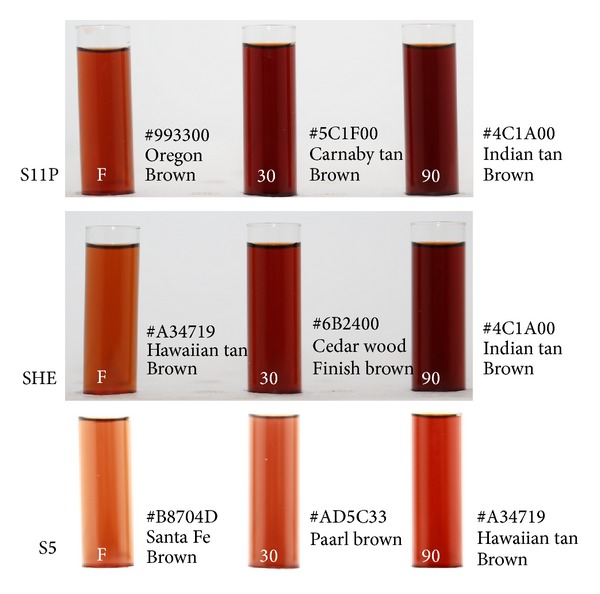
Showing periodical color change of the produced colloidal mixture by the tested isolates for up to 90 days. Colors are expressed in Hex (hexadecimal digit) values and verbal nomenclature using name your color project, available at http://chir.ag/projects/name-that-color/#B8704D.

The stability colloidal Ag nanoparticles synthesized by using different isolates (SHE, S11P, and S5) were observed optically by a color change and estimating the precipitation time (*τ*) (Figure 4). SHE and S11P did not lead to a significant change in color or appearance of agglomeration over a period of more than 90 days, while a precipitation of agglomerates using S5 was observed after 20 days, which indicates instability. Such observations were also checked using UV-vis, DLS, and TEM.

### 3.7. Zeta Potential Measurements

The long-term stability of colloidal Ag nanoparticles was monitored spectroscopically by zeta potential technique, which indicates the changes in surface charge with time. Such method is widely used to control the stability of colloidal metal nanoparticles. The metal nanoparticles with a large positive or negative zeta potential tend to repel each other and they do not show any disposition to come together. Nevertheless, in case of low absolute zeta potential values, these particles aggregate and flocculate due to the absence of repulsive force which prevents such agglomeration. Zeta potential results (*ζ*) of the freshly prepared colloidal AgNPs by using SHE, S11P, and S5 are shown to be having *ζ* values of −20, −14, and −30.4 mV, respectively. Table 1 represents the changes on the zeta potential values of different colloidal AgNPs samples prepared using various isolates at definite points of storage time. It can be seen from Table 1 that particles prepared using SHE and S11P were stable up to 90 days, and the zeta potential of these samples was somewhat constant within this time frame. These, nearly constant, values of zeta potential indicate a long-term stability of the corresponding colloids, which could be due to the production and excretion of microbial proteins that lead to stabilization of the nanoparticles. However, further studies are needed at both biochemical and molecular levels to obtain more understanding for this phenomenon. AgNPs obtained using S5 gave the highest value of zeta potential of −30 mV, which indicates a good stability. Surprisingly, this value was significantly decreased to −18.3 and −9.5 mV after 30 and 90 days, respectively. These values were found to be indications of instability, as further confirmed by UV-vis and TEM.

### 3.8. UV-Vis Spectroscopy

As shown earlier, the typical SP band position of colloidal AgNPs is in the visible light region between 350 nm and 600 nm. Table 1 provides information regarding the first examination of the stability of colloidal AgNPs obtained by SHE, S11P, and S5 isolates using different methods. It is noteworthy that for colloidal AgNPs prepared by SHE and S11P, no significant changes in the SP bands could be observed even after 90 days. This observation indicates that no aggregation took place, and hence the AgNPs formed using SHE and S11P was quite stable within this period of time. Conversely, the intensities of the SPRs progressively increase with time, which might be due to an increase in the number of AgNPs. On the contrary, colloidal AgNPs obtained by S5 as isolate did not form stable colloidal solutions, as proved by UV-vis (Table 1). The position of SPRs was broadened and red-shifted to longer wavelength (from 446 to 453 nm). This might point to the agglomeration of Ag particles with the long storage time.

### 3.9. TEM Investigation

The stability and the formation of aggregates colloidal AgNPs obtained with increasing the storage time can be precisely confirmed by TEM, whose results are in good agreement with zeta potential and UV-vis studies. The results achieved from TEM are summarized in Table 1. As we observed from the table, no considerable change in size, size distribution, and morphology between the fresh and stored samples were observed for AgNPs that occurred using SEM and S11P, as isolates. On the other hand, the particles aggregated with increasing ageing time and formed big clusters when S5 was used as isolate. Such large areas of agglomeration of AgNPs may be seen in Table 1. Therefore, we can conclude that the selection of isolate is very important for forming stable silver particles.

### 3.10. Antimicrobial Susceptibility Test

**Figure 5:**
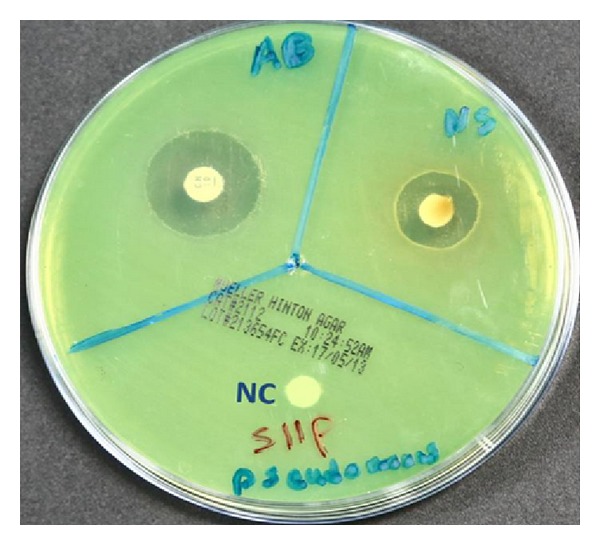
Zone of inhibition of silver nanoparticles produced by S11P against* P. aeruginosa* (ATCC 27853). NS: silver nanoparticles, AB: antibiotic of choice (gentamicin), and NC: negative control.

Table 2 shows the zones of inhibition produced by the selected three isolates representing the best active nanoparticles producer isolate* E. hermannii* (isolate SHE), medium active nanoparticles producer isolate* C. sedlakii* (isolate S11P), and the least* P. putida* (isolate S5). Isolate SHE showed that best antimicrobial activity against four of the tested pathogens, namely,* K. pneumonia*,* S. epidermidis*,* S. aureus*,* and E. coli.*, while isolate S11P showed the best antimicrobial activity only against* P. aeruginosa* (Figure 5). On the other hand, isolate S5 showed the least antimicrobial activity against all tested pathogens. Although the chosen antibiotics showed higher levels of activity against the tested pathogens, silver nanoparticles showed significant activity of inhibiting the growth of the pathogens.

Testing the antimicrobial efficacy against real pathogens showed that among* P. aeruginosa* isolates, sizes of the zones of inhibition produced by silver nanoparticles ranged between 10 and 35 millimeters with mean ± SD value of 23.7 ± 10.3, while sizes of the zones of inhibition produced by gentamicin ranged between 17 and 25 millimeters with mean ± SD values of 19.7 ± 2.9. Whereas, sizes of the zones of inhibition produced by combining silver nanoparticles and gentamicin ranged between 15 and 44 millimeters with mean ± SD values of 27.9 ± 8.7. Statistical analysis showed that there was no significant difference between treatment with silver nanoparticles and gentamicin against* P. aeruginosa* isolates with *P* value of 0.084, while, significant difference was observed between single treatments (silver nanoparticles or gentamicin) and silver nanoparticles and gentamicin combination with *P* values < 0.0001 in both cases. These results showed that silver nanoparticles treatment has better activity against some* P. aeruginosa* isolates compared with gentamicin antibiotic. It was found that silver nanoparticles showed the highest efficacy against five* P. aeruginosa* isolates (35 millimeters zone of inhibition) (22%). On the other hand, the highest efficacy observed for gentamicin (25 millimeters zone of inhibition) was observed against only one* P. aeruginosa* isolate (4.5%). In addition, a clear synergistic effect was observed between silver nanoparticles and gentamicin with zone of inhibition up to 44 millimeters. These results suggests the possibility of using silver nanoparticles and gentamicin combination to treat* P. aeruginosa* with lower probability of expected resistance, since no resistance so far has been reported against silver nanoparticles.

Furthermore, among the tested 21 DFU* S. aureus* isolates, sizes of the zones of inhibition produced by silver nanoparticles ranged between 8 and 12 millimeters with mean ± SD value of 9.5 ± 0.9, while sizes of the zones of inhibition produced by vancomycin ranged between 10 and 11.5 millimeters with mean ± SD value of 10.4 ± 0.5. Whereas, sizes of the zones of inhibition produced by combining silver nanoparticles and vancomycin ranged between 11 and 13 millimeters with mean ± SD value of 11.9 ± 0.5. Statistical analysis showed that there is significant difference between treatment with silver nanoparticles and vancomycin against* S. aureus* isolates with *P* value of 0.001. Moreover, significant difference was observed between single treatments (silver nanoparticles or vancomycin) and silver nanoparticles and gentamicin combination with *P* values < 0.0001 in both cases. Also, a clear synergistic effect was observed between silver nanoparticles and vancomycin against DFU* S. aureus* isolates.

**Figure 6:**
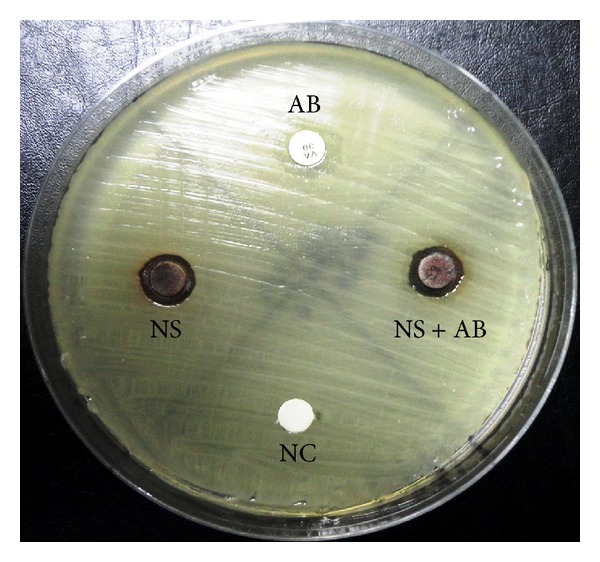
Zone of inhibition of silver nanoparticles produced by SHE against vancomycin resistant MRSA isolates collected from diabetic foot ulcer patients. NS: silver nanoparticles, AB: antibiotic of choice (vancomycin), NS + AB: silver nanoparticles and vancomycin, and NC: negative control.

**Figure 7:**
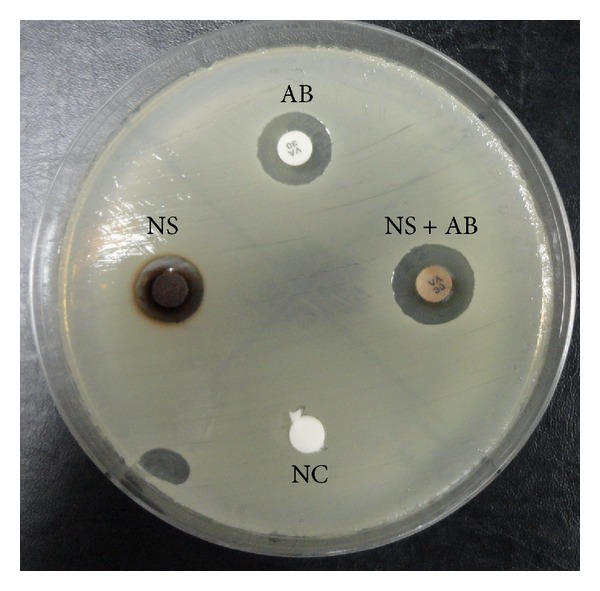
Synergistic effect between silver nanoparticles produced by SHE and vancomycin against MRSA isolates isolated from diabetic foot ulcer patients. NS: silver nanoparticles, AB: antibiotic of choice (vancomycin), NS + AB: silver nanoparticles and vancomycin, and NC: negative control.

For the tested 22 DFU MRSA isolates, sizes of the zones of inhibition produced by silver nanoparticles ranged between 6 and 11.5 millimeters with mean ± SD value of 9.6 ± 1.2, while sizes of the zones of inhibition produced by vancomycin ranged between 0 and 12.5 millimeters with mean ± SD value of 8.7 ± 4.9 (Figure 6). Whereas, sizes of the zones of inhibition produced by combining silver nanoparticles and vancomycin ranged between 8 and 14 millimeters with mean ± SD value of 12.2 ± 1.6. Statistical analysis showed that there was no significant difference between treatment with silver nanoparticles and vancomycin against MRSA isolates with *P* values of 0.346, while significant difference was observed between single treatments (silver nanoparticles or vancomycin) and silver nanoparticles and gentamicin combination with *P* values < 0.0001 in both cases. Our results surprisingly showed that silver nanoparticles produce successful antimicrobial effect against 5 MRSA isolates that were also resistant to vancomycin (MRSA-VRSA). Moreover, an obvious synergistic effect was observed between silver nanoparticles and vancomycin against MRSA isolates (Figure 7).

## 4. Conclusions

To the best of our knowledge, for the first time, silver nanoparticles were successfully obtained from isolates of* Escherichia hermannii*,* Citrobacter sedlakii,* and* Pseudomonas putida*. The produced colloidal AgNPs using these isolates exhibited single SPRs in the range of 430–450 nm. TEM investigations revealed that the AgNPs are ranged from 4 to 30 nm, depending on the nature of the used isolate and its produced metabolites. Zeta potential measurements confirmed that SHE and S11P gave the most stabile AgNPs. Of all these, SHE presented the best antimicrobial activity test against* K. pneumonia*,* S. epidermidis*,* S. aureus,* and* E. coli*. Meanwhile, isolate S11P showed the best antimicrobial activity only against* P. aeruginosa*. We showed that our silver nanoparticles exhibited equivalent or enhanced antimicrobial activity compared to gentamicin against* P. aeruginosa* isolates and vancomycin against* S. aureus* and MRSA isolates. In addition, we showed a clear synergistic effect between silver nanoparticles and tested antibiotics. These results suggest the possibility of the use of silver nanoparticles and selected antibiotics combination to treat fastidious infections.

## 5. Executive Summary

- Green synthesis, characterization, and antimicrobial activity of AgNPs were studied using three bacterial species, namely,* E. hermannii*,* C. sedlakii,* and* P. putida* isolated from arid Saudi Arabian environment.
- The synthesis of AgNPs was examined using different techniques such as UV-vis, zeta potential, and TEM. The measurement of changes in the surface plasmon band (SPB) position of AgNPs gave important information for particle size, shape, and size distribution.
- Stability and antimicrobial activity of AgNPs found to be dependent on the nature of bacterial species.* E. hermannii* showed the best antimicrobial activity against tested pathogens due to its small stable AgNPs.
- Our AgNPs presented the best antimicrobial activity test against* K. pneumonia*,* S. epidermidis*,* S. aureus, E. coli,* and* P. aeruginosa*.
- Our AgNPs exhibited equivalent or enhanced antimicrobial activity compared with drug of choice.
- A clear synergistic effect between AgNPs and tested antibiotics was shown.

## 6. Future Perspective 

Multidrug resistance is a rising problem in the treatment of infectious diseases. The wide use of broad-spectrum antibiotics has led to resistance to traditional antimicrobial agents for many bacterial human pathogens and has created a major threat to the global health care. AgNPs are considered as a potential source of novel antimicrobial agents, which offer numerous advantages such as broad-spectrum activity and lower tendency to induce resistance. In addition, broad-spectrum bioactivities of AgNPs make them potential agents in tackling serious problem of tumours and, particularly, multidrug resistant cancer cells. Moreover, AgNPs can be utilized in the diagnostics and treatment of different cancers. Therefore, this is an open area for many new studies in the cancer treatment with AgNPs. We also believe that in near future, AgNPs will have tremendous use as antiviral, antiprotozoal, and antiarthropod agents.

## Figures and Tables

**Table 1:** Characteristics and stability (*ζ* in millivolts) of the produced nanosilver particles after 90 days of production for the three selected isolates.

Isolates	Observation and color		*τ* (day)	*ζ* (mV_0_)	*ζ* (mV_90_)	Peak_max_	Size of Ag
	Fresh	Storage				UV-vis (nm)	TEM (nm)
SHE	Brown	Dark brown	Stable	−12.5	−22	440	4–12
S11P	Brown	Dark brown	Stable	−12.5	−15.9	442	4–15
S5	Light brown	Brown	20	−30	−9.5	453	4–30

**Table 2:** The 3 selected isolates SHE, S11P and S5, response to the produced nano particles and drug of choice against ATCC bacterial strains according to zone inhibition in millimeter.

Tested pathogenic strain	Tested antimicrobial agents			
	CRO	DA	CN	SN
Isolate SHE AgNPs				
*S. aureus *	20	25	NT	16
*S. epidermidis *	30	30	NT	17
*P. aeruginosa *	NT	NT	19	14
*E. coli *	29	NZI	NT	11
*K. pneumoniae *	29	NZI	NT	19

Isolate S11P AgNPs				
*S. aureus *	22	23	NT	12
*S. epidermidis *	3.2	3	NT	1.3
*P. aeruginosa *	NT	NT	19	15
*E. coli *	28	NZI	NT	10
*K. pneumoniae *	24	NZI	NT	14

Isolate S5 AgNPs				
*S. aureus *	20	25	NT	12
*S. epidermidis *	32	3	NT	11
*P. aeruginosa *	NT	NT	19	13
*E. coli *	29	NZI	NT	10
*K. pneumoniae *	29	NZI	NT	15

NT: not tested; NZI: no zone of inhibition was observed; CRO: ceftriaxone; DA: clindamycin; CN: gentamicin; SN: silver nanoparticles.

## Abbreviations

(NPs): Metal nanoparticles
(AgNPs): Silver nanoparticles
(SPRs): Surface plasmon resonances
(*ζ* values): Zeta potential values
(ATCC): American type culture collection
(EMB): Ethylene methylene blue medium
(SPB): Surface plasmon band
(DFU): Diabetic foot ulcer.
